# Stannous Source in Toothpastes Leads to Differences in Their Antimicrobial Efficacy

**DOI:** 10.3290/j.ohpd.b4424911

**Published:** 2023-09-22

**Authors:** Dominique Tobler, Olivier Braissant, Tuomas Waltimo, Michael M. Bornstein, Monika Astasov-Frauenhoffer

**Affiliations:** a Dentist, Department of Oral Health & Medicine, University Center for Dental Medicine Basel (UZB), University of Basel, Basel, Switzerland. Contributed to conceptualisation, validation, data curation, visualisation and original draft preparation.; b Senior Scientist, Center of Biomechanics and Biocalorimetry, University of Basel, c/o Department of Biomedical Engineering (DBE), Switzerland. Contributed to methodology and software, validation and data curation.; c Professor, Department of Oral Health & Medicine, University Center for Dental Medicine Basel (UZB), University of Basel, Basel, Switzerland. Contributed to validation, resources and data curation.; d Professor, Department of Oral Health & Medicine, University Center for Dental Medicine Basel (UZB), University of Basel, Basel, Switzerland. Contributed to conceptualisation, resources and supervision.; e Research Associate, Department Research, University Center for Dental Medicine Basel (UZB), University of Basel, Basel, Switzerland. Contributed to conceptualisation, methodology and software, validation, investigation, formal analysis, project administration and funding acquisition, data curation, visualisation and original draft preparation, supervision.; All authors have read and agreed to the submitted version of the manuscript.

**Keywords:** antimicrobial, biofilm, caries, stannous chloride, stannous fluoride, toothpaste

## Abstract

**Purpose:**

The aim of this in-vitro study was to investigate the antimicrobial efficacy of identical experimental toothpastes with different stannous sources.

**Materials and Methods:**

*Streptococcus mutans* biofilms were grown on protein-coated glass disks in static conditions for 24 h and thereafter exposed to toothpaste slurries or physiological saline (negative control; n = 15) for 30 s. Four experimental toothpastes were applied in this study, containing either stannous chloride (SnCl_2_; B: 3500 ppm Sn^2+^, and D: 3600 ppm Sn^2+^) or stannous fluoride (SnF_2_; C: 3500 ppm Sn^2+^, and E: 3600 ppm Sn^2+^). Marketed toothpaste meridol® (A: 3300 ppm SnF_2_) served as control. All five toothpastes contained amine fluoride (AmF). The biofilms were placed on agar surface and their metabolic activity was assessed by isothermal microcalorimetry over 96 h. The heat flow data was analysed for growth rate and lag time using grofit package in software R. Additionally, reduction of active biofilm compared to untreated control was calculated.

**Results:**

All toothpastes significantly prolong the lag time of treated biofilms in comparison to negative control (p < 0.05). Toothpastes containing SnF_2_ (C and E) prolonged the lag time statistically significantly compared to toothpastes containing SnCl_2_ (B and D) (p < 0.05). The maximum growth rate was statistically significantly reduced by all tested toothpastes compared to the untreated control group (p < 0.05). Toothpastes containing SnF_2_ (A, C and E) reached 59.9 ± 7.8, 61.9 ± 7.7, and 55.6 ± 7.0% reduction of active biofilm, respectively. Thus, they exhibit statistically significantly better results than toothpastes B (52.9 ± 9.9%) and D (44.7 ± 7.6%). Toothpaste D, which contains a slightly higher concentration of Sn^2+^, was the least effective in reducing active biofilm.

**Conclusion:**

The toothpastes containing SnF_2_ combined with AmF had the highest antimicrobial efficacy in this study.

Dental caries is a bacteria-mediated and fermentable carbohydrate-driven dynamic disease. The microflora of the oral cavity and plaque is challenged by the intake of sugars, which are fermented by the bacteria, thus leading to decreased pH in the oral environment. However, thanks to various mechanisms such as the buffering capacity of saliva, these short periods of lower pH do not lead to changes in plaque composition. Nevertheless, an increased frequency of sugar intake disrupts the homoeostasis of dental plaque, as it favours growth of acidogenic and aciduric bacteria which promote prolonged periods of low pH conditions and lead to caries. Commensal bacteria usually found in plaque are sensitive to decreased pH levels, leading to decreased growth under these conditions. Thus, an increased availability of carbohydrates causes an ecological shift in the microflora, which establishes conditions leading to caries development.^36^ *Streptococcus mutans* is one of the most commonly found bacteria in cariogenic biofilms, as it can easily form biofilms in conditions of low pH. Therefore, it is described as the primary pathogen in human dental caries.^15^ There are different factors that can affect the prevalence of caries, e.g., (a) individual factors (tooth morphology, saliva flow and buffering capacity, the composition oral microbiome), (b) behavioural factors (diet habits, especially the intake frequency and amount of fermentable carbohydrates, overall oral hygiene, medication, smoking), and (c) socioeconomic status and host genetics, as well as ameliorating factors (e.g., fluoride) which facilitate remineralisation.^32^

For caries prevention, mechanical control of oral biofilm on tooth surfaces has become indispensable. However, this requires skill and compliance and is not always effective in the interdental areas. Therefore, research on the development of new antimicrobial ingredients and re-assessment of those which already exist in supporting oral-care products such as toothpastes is of great importance.^30,36^

Fluoride can alter the plaque microbiota, change its metabolism and consequently reduce cariogenic microbiota.^41^ Moreover, topical fluoride application elevates the fluoride levels in oral fluids, which supports the process of remineralisation in enamel.^39^ Several fluoride compounds are used in toothpastes at various concentrations, such as amine fluoride (AmF), sodium fluoride (NaF), sodium monofluorophosphate (Na_2_PO_3_F) or stannous fluoride (SnF_2_).^22^ Due to the greater affinity of hydrophilic counterions to the enamel surface, AmF is associated with reduction of plaque adhesiveness and is shown to exhibit longer clearance in the oral cavity and dental plaque; thus, it has a pronounced activity on plaque. Over a period of 3 – 6 h, AmF is strongly glycolytic and has profound bacteriostatic and bactericidal effects.^9^ While the antimicrobial effect of NaF consists in inhibiting the growth of oral bacteria, their acid and glucan production,^44^ Na_2_PO_3_F interacts with and inhibits the activity of enzymes produced by caries-related bacteria, thereby suppressing their growth.^26^

Although research on SnF_2_ in oral hygiene products has been ongoing since the mid-1950s, its efficacy could be enhanced by revealing more information about different concentrations and formulations. The antibacterial potential is promising, as in-vivo studies show statistically significantly greater caries reduction as well as less plaque and gingivitis when using stannous fluoride (SnF_2_) compared to sodium fluoride (NaF).^20,29,43^ On the one hand, the antimicrobial effect is achieved by tin accumulation in plaque.^27^ On the other hand, SnF_2_ dentifrices seem to target metabolic functions of bacteria and thereby inhibit their enzymes, including acid and glucan production.^44^ Additionally, the remineralisation process is supported, as CaF_2_ precipitates are formed on the enamel surface, which are capable of subsequently releasing fluorine particles.^13^ The formation of precipitates that obstruct dentin tubules also results in hypersensitivity relief.^16,33^ Long-term application of oral hygiene products containing SnF_2_ results in a reduction of pathogenic oral bacteria, such as *Streptococcus* species, as well as in an increase in health-associated bacteria.^20^ Stannous chloride (SnCl_2_) is a commonly used active ingredient in toothpastes, as it contains both anti-plaque and anti-gingivitis properties. It forms a complex with proteins in saliva and on tooth surfaces, which can disrupt the formation of plaque and prevent bacterial adhesion to tooth surfaces.^19^ However, it has been reported that SnCl_2_ shows less tin accumulation in plaque than SnF_2_ and is therefore suggested to have less potent bacteriostatic and bactericidal effects against *S. mutans*.^12,40^ Nevertheless, there is still need for more information about the effect of the stannous source as an antimicrobial agent in toothpaste.^31^

Isothermal microcalorimetry (IMC) is a culture-based method that allows detection of the antibacterial activity in vitro by measuring heat production rates. Although it is a simple method, it has some very relevant advantages. The method measures the metabolic activity of the biofilm as an entity without disturbing its structure on solid agar surfaces.^7^ The sensitivity of modern microcalorimeters is higher than that of many conventional methods, such as spectrophotometry or enzymatic assays, as it is able to detect the metabolic heat production of 10^4^ – 10^5^ bacteria. Moreover, the accuracy IMC provides for dynamic measurements cannot be achieved with microscopic enumeration, plate counts or protein assays.^8^

Thus, the aim of the study was to investigate the antimicrobial potential of four toothpastes, which differ in their stannous source (SnF_2_ or SnCl_2_) and concentration but not in fluoride concentration that was for all tested samples 1400 ppm. A commercially available toothpaste, meridol (Colgate-Palmolive Europe sàrl; Therwil, Switzerland), containing SnF_2_ in combination with AmF, was used as a control as it has multiple proven beneficial effects on plaque reduction and gingival health.^27^ The null hypothesis of this study was that toothpastes show the same efficacy regardless of their stannous source.

## Materials and Methods

### Biofilm Formation and Treatment

**Fig 1:**
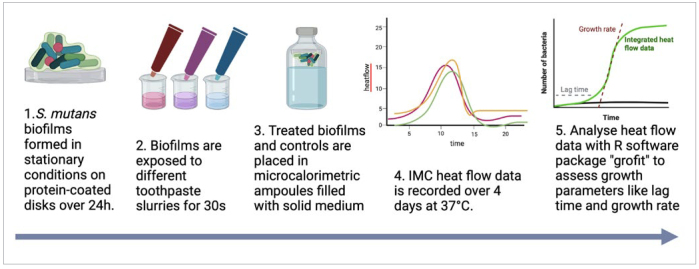
Flow diagram illustrating the main steps of the experiment: (1) biofilm formation on salivary-protein-coated disks; (2) exposure to toothpaste slurries; (3) measurement of metabolic activity with isothermal microcalorimetry (IMC); (4) heatflow data of IMC; (5) analysis of IMC data to assess growth parameters (lag time and growth rate) of the biofilms.

The main steps of the workflow of the experiment are illustrated in Fig 1. An aliquot of 10 µl of *S. mutans* (ATCC 25175) stock solution was inoculated on Columbia blood agar plate (BBL Columbia Agar Base, BD; Allschwil, Switzerland) and incubated for 48 h at 37°C. Thereafter, one colony was selected and suspended to 25 ml of Todd Hewitt (TH) media (Bacto Todd Hewitt broth, BD) supplemented with 0.5% sucrose (D(+) sucrose, Fluka; Buchs, Switzerland). The culture was incubated for 22 h at 37°C. The grown bacteria were harvested by centrifugation (8500 rpm, 5 min, RT; Sigma 4-16KS, Kuhner; Basel, Switzerland) and resuspended in simulated body fluid (SBF; 7.996 g NaCl, 0.35 g NaHCO_3_, 0.224 g KCl, 0.228 g K_2_HPO_4_ x 3H_2_O, 0.305 g MgCl_2_ x 6H_2_O, 0.278 g CaCl_2_, 0.071 g Na_2_SO_4_, 6.057 g (CH_2_OH)_3_CNH_2_ dissolved in 1 l of ultra-pure water, pH adjusted to 7.25 with 1 mol/l HCl, all chemicals from Sigma Aldrich; Buchs, Switzerland)^10^ supplemented with 10% TH medium and 1% sucrose.

Meanwhile, 5-mm glass disks (Biosystems Switzerland AG, Muttenz, Switzerland) were coated with sterile pooled saliva mixture for 15 min.^3^ Briefly, the saliva was collected anonymously from healthy volunteers by paraffin stimulation. After collection, it was ultrasonicated for 30 s (30 W, Vibracell, Sonics & Materials; Newtown, CT, USA) and filtered through 70-µm filters (Cell Strainer, Becton-Dickinson; Basel, Switzerland), followed by 40 min centrifugation at 22,000 g at 4°C. Then the supernatant was sterilised by using two connected filters (0.45 and 0.22 μm; Millex-HV and Millex-GV, respectively, Millipore; Schaffhausen, Switzerland). Pooled samples were stored in aliquots at -20°C.

Thereafter, the protein-coated disks were placed in 24-well plates (Sarstedt; Sevelen, Switzerland). Then, 1 ml of freshly prepared bacterial suspension and 0.5 ml of TH medium were added to each well, followed by incubation of the disks for 24 h at 37°C.

Subsequently, the disks were dipped in 0.9% NaCl three times to remove loosely adhered cells, after which they were placed in 1 ml of five freshly-prepared toothpaste slurries (23% of the original solution) for 30 s (n = 5). Details on the stannous source and concentrations are given in Table 1. The biofilms serving as untreated negative controls were exposed to 0.9% NaCl for 30 s (n = 5).

**Table 1:** Commercially available toothpaste (meridol) and the four experimental toothpastes (B – E) used in this study, with stannous source and concentration

Toothpaste	Fluoride [ppm]	Sn(II) [ppm]	AmF	SnF_2_	SnCl_2_
Toothpaste A (meridol)	1400	3300	x	x	
Toothpaste B	1400	3500	x		x
Toothpaste C	1400	3500	x	x	
Toothpaste D	1400	3600	x		x
Toothpaste E	1400	3600	x	x	

The test toothpaste slurries were freshly prepared for each run as follows: 3 g of the assigned test toothpaste was weighed into a 50 ml tube, then the tube was filled with sterile milliQ water to a total weight of 13 g. After thoroughly closing the centrifuge tube, it was placed on a Vortex mixer (2800 rpm, 45 s) until it became a homogenous slurry.

After the toothpaste slurry treatment, the disks were placed in 2 ml of inactivation solution (1 g trypton and 8.5 g NaCl dissolved in 1 L H_2_O, to which 1 g L-histidine, 5 g Na_2_S_2_O_3_, 3 g lecithin and 94 ml Tween-80 were added; all Sigma-Aldrich). Thereafter, the disks were again dipped in 0.9% NaCl three times and placed in IMC ampoules containing 3 ml of Columbia blood agar. The experiment was repeated a total of three times, resulting in 15 samples per each treated group as well as for the untreated group.

### Isothermal Microcalorimetry (IMC)

IMC ampoules filled with 3 ml Columbia blood agar were prepared and disks with treated biofilms as well as untreated controls were placed on the agar with the biofilm facing the agar. The IMC ampoules were closed in aerobic conditions and placed in a TAM 48 microcalorimeter (TA Instruments; New Castle, DE, USA), which measured and recorded the metabolic activity of the biofilms at 37°C for up to 96 h.

The heat-flow data obtained over time with IMC was analysed for growth rate (µ; 1/h) and lag time (l; h) by fitting the heat-over-time curve (i.e., resulting from the integration of the heat-flow curve) with Gompertz’s equation, using the “grofit” package^18^ in R statistical software (R Foundation for Statistical Computing, Vienna, Austria), as described earlier.^3^

The reduction of the biofilm population was estimated by using the following equation


Inhibition=100−100(sampleλ−controlλln(2)controlλ)2

(1)

### Statistical Analysis

The Shapiro-Wilk normality test was applied to the samples and differences between toothpastes and untreated controls were assessed by Student’s t-test with significance set to p < 0.05. Differences between the different acids and amino bases were assessed by conducting a one-way ANOVA using GraphPad Prism (version 9.3.1 for Mac, GraphPad Software; La Jolla, CA, USA, www.graphpad.com).

## Results

### Differences in Parameter Lag Time (Fig 2a)

**Fig 2:**
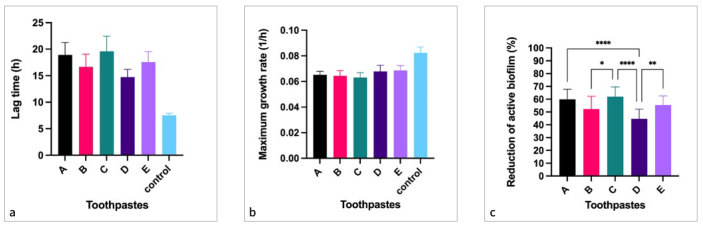
Five toothpastes (A-E) which differ in their stannous source were examined for their antimicrobial properties. Three different parameters were assessed from IMC data: (A) lag time in h, (B) maximum growth rate in 1/h, and (C) reduction of active biofilm in % compared to untreated control biofilm. Significant differences between groups are indicated with asterisks (* p < 0.05, ** p < 0.01, **** p < 0.001).

The lag times of *S. mutans* suspension treated with toothpastes A-E, which differ in their stannous source and concentrations (Table 1), are shown in Fig 2a. All toothpastes statistically significantly prolonged the lag time of treated biofilms in comparison to untreated control biofilm (p < 0.05). There was no difference in efficacy when comparing the commercially-available toothpaste meridol (A) to the experimental toothpastes C and E, which contained SnF_2_ as a stannous source (p > 0.05). In contrast, toothpastes B and D, containing SnCl_2_ as a stannous source, showed a statistically significantly shorter lag time (p < 0.05) compared to meridol®. Toothpaste D (3600 ppm SnCl_2_) was the least effective, showing a statistically significantly (p < 0.05) smaller increase of the lag time compared to all other toothpastes, except for toothpaste B (3500 ppm SnCl_2_). In this combination, the higher amount of Sn^2+^ did not statistically significantly affect the efficacy of the toothpaste (p > 0.05).

### Differences in Parameter Maximum Growth Rate (Fig 2b)

Figure 2b shows the maximum growth rate of *S. mutans* biofilms treated with the toothpastes A-E and the untreated control biofilms. The maximum growth rate was statistically significantly lowered by all tested toothpastes compared to the untreated control group (p < 0.05). When comparing different treated groups, only toothpaste C showed a slightly statistically significant reduction of growth rate in comparison to toothpastes D and E (p < 0.05); all other groups showed no differences (p > 0.05).

### Reduction of Active Biofilm (Fig 2c)

The reduction of active biofilm achieved by toothpastes A-E is shown in Fig 2c and given as a percentage (calculated as shown in equation 1). The results resemble the pattern of Fig 2a. Toothpastes containing SnF_2_ (A, C and E) reach 59.9 ± 7.8, 61.9 ± 7.7, 55.6 ± 7.0% reduction of active biofilm, respectively. Therefore, they exhibit statistically significantly better results than toothpastes B (52.9 ± 9.9%) and D (44.7 ± 7.6%) (p < 0.05). Toothpaste D, which contains a slightly higher concentration of SnCl_2_, was the least effective in reducing active biofilm.

## Discussion

In the present in-vitro study, different antimicrobial properties of four experimental toothpastes containing AmF (1400 ppm fluoride) combined with tin either derived from SnF_2_ (3500 ppm Sn^2+^ and 3600 ppm Sn^2+^) or from SnCl_2_ (3500 ppm Sn^2+^ and 3600 ppm Sn^2+^) were investigated in a cariogenic model biofilm using *S. mutans*. In addition to the experimental toothpastes, the commercially available toothpaste meridol, containing both AmF and SnF_2_ (3300 ppm Sn^2+^), was examined.

The experiment was carried out using slurries of toothpastes without brushing. Therefore, the antibacterial effect of the active ingredients was assessed without the mechanical cleaning effect present during normal toothbrushing. Similar procedures have been used previously to assess the efficacy of toothpastes.^1,42^ Although stannous agents are being used in oral hygiene products, knowledge about their antimicrobial effect in different concentrations and combinations in otherwise identical toothpastes is limited.

Toothpastes tested containing a stannous source derived from SnF_2_ (toothpastes A, C and E) prolonged the lag time and yielded greater reduction of active biofilm than did toothpastes containing tin derived from SnCl_2_ (toothpastes B and D). Moreover, results for the toothpastes containing SnCl_2_ in this study are consistent with limited reports in the literature, which do not demonstrate efficacy for this active combination as an antimicrobial agent.^6^ Rather, SnCl_2_ in connection with oral hygiene products has mainly been studied for its anti-erosive potential in enamel and dentin.^11,14,23^ Toothpastes with SnCl_2_ have shown a statistically significantly higher plaque reduction than comparable dentifrices with anti-erosion properties.^6^ In other studies on erosive mineral loss, a synergistic effect between fluoride and tin has been described when both were present in a solution together (either as SnF_2_ or as AmF/SnCl_2_); the inhibition of mineral loss was statistically significantly greater.^2,24,34^

The same synergistic effect of SnF_2_ and AmF applies to the antimicrobial activity; it has been confirmed that the antibacterial activity is greater when fluoride is associated with the cation Sn^2+^ or amine.^35^ The previously described higher bioavailability of stannous ions in the presence of fluoride might explain this, but the evidence is relatively limited.^12,30^ However, there seems to be a difference when comparing the stannous source, i.e., toothpastes B and D (SnCl_2_) vs toothpastes A, C and E (SnF_2_), as the data show a slightly lower bactericidal (based on delay in lag time due to initially surviving inoculum) and bacteriostatic (based on reduction in growth rate) effect. The reduction of bacterial biomass/virulence and inhibition of bacterial metabolism is achieved by the Sn^2+^ ion, which has been shown to be the bioactive species exerting the antiplaque effect.^28^ The correlation of stabilised Sn^2+^ to antibacterial performance is linear, whereas the proportion of Sn^4+^ (oxidation state) is positively correlated with bacterial viability and therefore inactive against bacteria. The challenge of stabilising SnF_2_ needs to be overcome; reportedly, the formulations of SnF_2_ and AmF stabilise the positive properties of SnF_2_ as well.^25,35,37^

Moreover, the antibacterial potential of tin seems to be dependent on the concentration, but not in a dose-dependent way. Toothpastes with SnF_2_ (e.g., toothpaste C) contain 3500 ppm Sn^2+^ and showed higher efficacy against the tested biofilms than did toothpaste A, which contains 3300 ppm Sn^2+^, and toothpaste E, with 3600 ppm Sn^2+^. The exact same phenomenon applies to the toothpastes containing SnCl_2_: toothpaste B, containing 3500 ppm Sn^2+^, shows higher antimicrobial activity than the higher concentrated toothpaste D, containing 3600 ppm Sn^2+^. Unfortunately, evidence of synergy being involved in the antimicrobial effect is currently scarce. Overall, this solution seems to be understudied, which is probably due to its propensity to cause extrinsic stains and its bad taste.^17^ This has been overcome by new formulations of SnF_2_- dentifrices, for example, Colgate TotalSF, containing 0.454% SnF_2_ and 1% zinc phosphate, which provides statistically significantly higher stain reduction compared to a competitor paste (26%) and SnF_2_ gel (35%).^21^ Further, using sodium hexametaposphate with SnF_2_ formulations also removes extrinsic stains and provide long-lasting inhibition of new-stain chromogen adsorption.^5^

Nevertheless, as the oral cavity is more complex than the biofilm model used in the present study, the findings should be interpreted with caution. Additionally, the roughness of the surface used in this study resembles that of enamel, but hydroxyapatite or dental enamel would be better for representing the real cariogenic situation. Nonetheless, the surface here was covered with salivary pellicle and measuring de- and remineralisation was not in the scope of this study. It is important to note that the use of simplified models can be considered as a limitation; however, it is cost effective and helps to screen for optimal or promising formulations in early stages of development. Additionally, the current study only assessed the antimicrobial effect of the toothpastes based on their stannous source and combination with AmF, but did not investigate the real conditions of brushing, in which biofilm is partially removed mechanically and the penetration of the toothpaste into the deeper layers of biofilm is improved.^4^ The limited number of replicates combined with a high number of groups used in the statistical analysis could be considered a limitation of this study; thus, a retrospective power analysis was performed. It showed that statistical analysis was sufficiently powered (power > 0.99) to detect differences between the controls and other groups. Smaller differences (<10% of the measured values) between treatment groups should, however, be considered with care.

## Conclusions

The null hypothesis was rejected, as toothpastes with SnCl_2_ were less efficaceous than toothpastes with SnF_2_. Toothpastes containing SnF_2_ as the stannous source showed an increase in antibacterial properties when concentrations increased from 3300 ppm Sn^2+^ to 3500 ppm Sn^2+^. However, at a higher concentration (3600 ppm Sn^2+^), the efficacy did not increase.

Concerning toothpastes containing SnCl_2_, the antimicrobial efficacy decreased when the concentration of Sn^2+^ increased from 3500 ppm Sn^2+^ to 3600 ppm Sn^2+^. The highest antimicrobial efficacy was reached with the stannous source of 3500 ppm SnF_2_.

## References

[ref1] Arweiler NB, Auschill TM, Reich E, Netuschil L (2002). Substantivity of toothpaste slurries and their effect on reestablishment of the dental biofilm. J Clin Periodontol.

[ref2] Arweiler NB, Müller-Breitenkamp F, Heumann C, Laugisch O, Auschill TM (2021). Antibacterial action, substantivity and anti-plaque effect of different toothpaste slurries – a randomised controlled trial. Oral Health Prev Dent.

[ref3] Astasov-Frauenhoffer M, Braissant O, Hauser-Gerspach I, Weiger R, Walter C, Zitzmann NU (2014). Microcalorimetric determination of the effects of amoxicillin, metronidazole, and their combination on in vitro biofilm. J Periodontol.

[ref4] Auschill TM, Deimling D, Hellwig E, Arweiler NB (2007). Antibacterial effect of two toothpastes following a single brushing. Oral Health Prev Dent.

[ref5] Baig A, He T, Buisson J, Sagel L, Suszcynsky-Meister E, White DJ (2005). Extrinsic whitening effects of sodium hexametaphosphate – a review including a dentifrice with stabilized stannous fluoride. Compend Contin Educ Dent.

[ref6] Bellamy PG, Prendergast M, Strand R, Yu Z, Day TN, Barker ML (2011). Can anti-erosion dentifrices also provide effective plaque control?. Int J Dent Hyg.

[ref7] Braissant O, Bachmann A, Bonkat G (2015). Microcalorimetric assays for measuring cell growth and metabolic activity: methodology and applications. Methods.

[ref8] Braissant O, Wirz D, Gopfert B, Daniels AU (2010). Use of isothermal microcalorimetry to monitor microbial activities. FEMS Microbiol Lett.

[ref9] Brecx M (1997). Strategies and agents in supragingival chemical plaque control. Periodontol 2000.

[ref10] Cho SB, Nakanishi K, Kokubo T, Soga N, Ohtsuki C, Nakamura T (1995). Dependence of apatite formation on silica gel on its structure: effect of heat treatment. J Am Ceram.

[ref11] Clark-Perry D, Levin L (2020). Comparison of new formulas of stannous fluoride toothpastes with other commercially available fluoridated toothpastes: A systematic review and meta-analysis of randomised controlled trials. Int Dent J.

[ref12] Ferretti GA, Tanzer JM, Tinanoff N (1982). The effect of fluoride and stannous ions on Streptococcus mutans. Viability, growth, acid, glucan production, and adherence. Caries Res.

[ref13] Fiorillo L, Cervino G, Herford AS, Laino L, Cicciu M (2020). Stannous fluoride effects on enamel: a systematic review. Biomimetics (Basel).

[ref14] Ganss C, Lussi A, Sommer N, Klimek J, Schlueter N (2010). Efficacy of fluoride compounds and stannous chloride as erosion inhibitors in dentine. Caries Res.

[ref15] Gross EL, Beall CJ, Kutsch SR, Firestone ND, Leys EJ, Griffen AL (2012). Beyond Streptococcus mutans: dental caries onset linked to multiple species by 16S rRNA community analysis. PLoS One.

[ref16] He T, Barker ML, Biesbrock AR, Miner M, Qaqish J, Sharma N (2014). A clinical study to assess the effect of a stabilized stannous fluoride dentifrice on hypersensitivity relative to a marketed sodium fluoride/triclosan control. J Clin Dent.

[ref17] Horst JA, Tanzer JM, Milgrom PM (2018). Fluorides and other preventive strategies for tooth decay. Dent Clin North Am.

[ref18] Kahm M, Hasenbrink G, Lichtenberg-Fraté H, Ludwig J, Kschischo M (2010). grofit: fitting biological growth curves with R. J Stat Softw.

[ref19] Kirsch J, Hannig M, Winkel P, Basche S, Leis B, Pütz N, Kensche A, Hannig C (2019). Influence of pure fluorides and stannous ions on the initial bacterial colonization in situ. Sci Rep.

[ref20] Kruse AB, Schlueter N, Kortmann VK, Frese C, Anderson A, Wittmer A (2021). Long-term use of oral hygiene products containing stannous and fluoride ions: effect on viable salivary bacteria. Antibiotics (Basel).

[ref21] Li Y, Suprono M, Mateo LR, Zhang YP, Denis J, D’Ambrogio R (2019). Solving the problem with stannous fluoride: Extrinsic stain. J Am Dent Assoc.

[ref22] Lippert F (2013). An introduction to toothpaste – its purpose, history and ingredients. Monogr Oral Sci.

[ref23] Lorenz K, Hoffmann T, Heumann C, Noack B (2019). Effect of toothpaste containing amine fluoride and stannous chloride on the reduction of dental plaque and gingival inflammation. A randomized controlled 12-week home-use study. Int J Dent Hyg.

[ref24] Lussi A, Carvalho TS (2015). The future of fluorides and other protective agents in erosion prevention. Caries Res.

[ref25] Mallatt M, Mankodi S, Bauroth K, Bsoul SA, Bartizek RD, He T (2007). A controlled 6-month clinical trial to study the effects of a stannous fluoride dentifrice on gingivitis. J Clin Periodontol.

[ref26] Marinho VC, Higgins JP, Logan S, Sheiham A (2003). Fluoride toothpastes for preventing dental caries in children and adolescents. Cochrane Database Syst Rev.

[ref27] Mayhew RR, Brown LR (1981). Comparative effect of SnF_2_, NaF, and SnCl_2_ on the growth of Streptococcus mutans. J Dent Res.

[ref28] Myers CP, Pappas I, Makwana E, Begum-Gafur R, Utgikar N, Alsina MA, Fitzgerald M, Trivedi HM, Gaillard JF, Masters JG, Sullivan RJ (2019). Solving the problem with stannous fluoride: Formulation, stabilization, and antimicrobial action. J Am Dent Assoc.

[ref29] Ogaard B, Alm AA, Larsson E, Adolfsson U (2006). A prospective, randomized clinical study on the effects of an amine fluoride/stannous fluoride toothpaste/mouthrinse on plaque, gingivitis and initial caries lesion development in orthodontic patients. Eur J Orthod.

[ref30] Paraskevas S (2005). Randomized controlled clinical trials on agents used for chemical plaque control. Int J Dent Hyg.

[ref31] Rajendiran M, Trivedi HM, Chen D, Gajendrareddy P, Chen L (2021). Recent development of active ingredients in mouthwashes and toothpastes for periodontal diseases. Molecules.

[ref32] Rosier BT, De Jager M, Zaura E, Krom BP (2014). Historical and contemporary hypotheses on the development of oral diseases: are we there yet?. Front Cell Infect Microbiol.

[ref33] Schiff T, He T, Sagel L, Baker R (2006). Efficacy and safety of a novel stabilized stannous fluoride and sodium hexametaphosphate dentifrice for dentinal hypersensitivity. J Contemp Dent Pract.

[ref34] Schlueter N, Hardt M, Lussi A, Engelmann F, Klimek J, Ganss C (2009). Tin-containing fluoride solutions as anti-erosive agents in enamel: an in vitro tin-uptake, tissue-loss, and scanning electron micrograph study. Eur J Oral Sci.

[ref35] Shapira L, Shapira M, Tandlich M, Gedalia I (1999). Effect of amine fluoride-stannous fluoride containing toothpaste (Meridol) on plaque and gingivitis in adults: a six-month clinical study. J Int Acad Periodontol.

[ref36] Sjögren K (2001). How to improve oral fluoride retention?. Caries Res.

[ref37] Steiger J, Braissant O, Waltimo T, Astasov-Frauenhoffer M (2021). Efficacy of experimental mouth rinses on caries-related biofilms in vitro. Front Oral Health.

[ref38] Takahashi N, Nyvad B (2011). The role of bacteria in the caries process: ecological perspectives. J Dent Res.

[ref39] ten Cate JM, Featherstone JDB (1991). Mechanistic aspects of the interactions between fluoride and dental enamel. Crit Rev Oral Biol Med.

[ref40] Tinanoff N, Camosci DA (1980). Microbiological, ultrastructural and spectroscopic analyses of the anti-tooth-plaque properties of fluoride compounds in vitro. Arch Oral Biol.

[ref41] Van Loveren C (2001). Antimicrobial activity of fluoride and its in vivo importance: identification of research questions. Caries Res.

[ref42] Weiland B, Netuschil L, Hoffmann T, Lorenz K (2008). Substantivity of amine fluoride/stannous fluoride following different modes of application: a randomized, investigator-blind, placebo-controlled trial. Acta Odontol Scand.

[ref43] White DJ (2007). Effect of a stannous fluoride dentifrice on plaque formation and removal: a digital plaque imaging study. J Clin Dent.

[ref44] Zameck RL, Tinanoff N (1987). Effects of NaF and SnF_2_ on growth, acid and glucan production of several oral streptococci. Arch Oral Biol.

